# Improper Light Curing of Bulkfill Composite Drives Surface Changes and Increases *S. mutans* Biofilm Growth as a Pathway for Higher Risk of Recurrent Caries around Restorations

**DOI:** 10.3390/dj9080083

**Published:** 2021-07-30

**Authors:** Haifa Maktabi, Maria Salem Ibrahim, Abdulrahman A. Balhaddad, Qoot Alkhubaizi, Isadora Martini Garcia, Fabrício Mezzomo Collares, Howard Strassler, Ana Paula P. Fugolin, Carmem S. Pfeifer, Mary Anne S. Melo

**Affiliations:** 1 Division of Operative Dentistry, Department of General Dentistry, University of Maryland School of Dentistry, Baltimore, MD 21201, USA; hmaktabi@umaryland.edu (H.M.); qootalkhubaizi@umaryland.edu (Q.A.); howrdstrassler@umaryland.edu (H.S.); 2 Ph.D. Program in Biomedical Sciences, University of Maryland School of Dentistry, Baltimore, MD 21201, USA; mibrahim@umaryland.edu (M.S.I.); aabalhaddad@umaryland.edu (A.A.B.); 3 Department of Preventive Dental Sciences, College of Dentistry, Imam Abdulrahman Bin Faisal University, Dammam 31411, Saudi Arabia; 4 Department of Restorative Dental Sciences, College of Dentistry, Imam Abdulrahman Bin Faisal University, Dammam 31411, Saudi Arabia; 5 Dental Materials Laboratory, School of Dentistry, Federal University of Rio Grande do Sul, Rua Ramiro Barcelos, 2492, Rio Branco, Porto Alegre 90035-003, Brazil; isadora.mgarcia@hotmail.com (I.M.G.); fabricio.collares@ufrgs.br (F.M.C.); 6 Division of Biomaterials and Biomechanics, Department of Restorative Dentistry, School of Dentistry, Oregon Health & Science University, Portland, OR 97239, USA; fugolin@ohsu.edu (A.P.P.F.); Carmenspfeifer@ohsu.edu (C.S.P.)

**Keywords:** composite resins, polymerization, biofilms, curing lights, dental, surface properties

## Abstract

How dentists cure a resin-based material has deleterious effects on the material’s properties and its interaction with surrounding dental tissues. Biofilm accumulation has been implicated in the pathogenesis of carious lesions around dental restorations, with its composition manifesting expressed dysbiosis in patients suffering from dental caries. To evaluate the influence of varying radiant exposure on the degree of conversion (DC%), *Streptococcus mutans* biofilm growth, and surface roughness of bulk-fill composites under different light-curing conditions. Two light-curing units (LCU) at 600 and 1000 mW/cm^2^ were used to simulate curing conditions with different angulations (∢20° and ∢35°) or 2 mm-distance displacements of the LCU tip. The radiant exposure (RE) was assessed, and the composites were analyzed for DC%. Biofilm formation was induced over the bulk-fill composites and analyzed via colony-forming units counting and scanning electron microscopy (SEM). The surface roughness was analyzed via a profilometer and SEM after biofilm formation. Curing conditions with different angulation or displacement decreased RE compared to the “optimal condition”. The moderately (∢35°) angulated LCU tip and low (600 mW/cm^2^) radiant emittance significantly reduced the DC% (*p* < 0.05). The difference in DC% between the top and bottom of the composites ranged from 8 to 11% for 600 mW/cm^2^ and 10 to 20% for 1000 mW/cm^2^. Greater *S. mutans* biofilm and surface changes were found in composites with non-optimal RE delivery (e.g., tip displacement and angulation) (*p* < 0.05). Inadequate polymerization of bulk-fill composites was associated with more biofilm accumulation and surface topography changes. Overall, non-optimally performed curing procedures reduced the amount of delivered RE, which led to low DC%, more biofilm formation, and higher surface roughness. The improper light-curing of bulk-fill composites compromises their physicochemical and biological properties, which could lead to inferior clinical performance and reduced restorative treatments’ longevity.

## 1. Introduction

Appropriated photoactivation that enables optimal curing and cross-linking of polymer chains is essential for a reliable dental resin behavior [1,2]. Photopolymerization may become even more critical for clinical success with the increasing use of bulk-fill composites presenting different photoinitiators, monomer composition, and filler content [3]. Bulk-fill composites underpin modern restorative dentistry. This class of composites was developed to overcome limitations associated with conventional resin-based composites (RBCs), such as limited depth of cure and polymerization shrinkage [4,5].

Bulk-fill composites allow placement in increments up to 4–5 mm thick, which can speed up the restorative procedure, minimize oral fluid contamination, and eliminate the potential for voids between layers [6]. On the other hand, conventional RBCs should be limited to 2 mm increments to allow adequate light transmittance [7]. The light transmittance differs between these two types of composites. Bulk-fill composite is more translucent and has a higher depth of cure than conventional RBCs [8,9]. The light transmittance up to 4 mm can also be attributed to a different photoinitiator incorporated into the bulk-fill composite system [9]. Moreover, more modern bulk-fill composites undergo RAFT (reversible addition–fragmentation chain transfer) polymerization resulting in more cross-linking and improved depth of cure [10].

In compliance with the particular set of strategies developed to enhance the depth of bulk-fill cure, proper curing of bulk-fill composites is also essential for suitable service life in the oral environment [11,12,13,14]. The curing of bulk-fill demands to be optimal and may be compromised by improper curing practices [15,16,17,18]. If the bulk-fill is not adequately cured, the bottom layer, usually 4 mm or more distant from the light-curing tip, is the most affected [19]. This condition can be particularly significant as it can constitute the bottom layer in contact with the gingival wall in proximal cavity preparation for most class II restorations [20]. It is worth noting that the bottom layer in the above-described scenario is exposed to the oral environment and represents an area of constant concern to operative dentists.

Previous studies have emphasized that uncured monomers can be leach out, increasing the adhesion and growth of cariogenic species [17,21,22]. Thus, the enrichment of aciduric and acidogenic species, such as *Streptococcus mutans*, and the uncured rates of monomers can be factors governing the degradation behavior of the resin blend. This degradation may facilitate gap formation with bacterial penetration and subsequently increase the risk of developing secondary caries [23,24].

One of the most important parameters influencing the optimal curing is the delivery of radiant exposure (R_E_). This parameter refers to the amount of radiant energy absorbed by the restoration to achieve highly cross-linked networks [11,12]. Therefore, the curing adequacy often relies on the degree of conversion varying from 50 to 70% [25].

The majority of prior literature has also considered the radiance emittance (mW/cm^2^) generated by the light-curing unit (LCU) as essential to polymerize the composite optimally [14]. However, LCU with low radiant emittance or improper maintenance could compromise the delivered R_E_ (J/cm^2^) to a bulk-fill composite [19].

Furthermore, both of these factors are greatly influenced by the curing conditions. Some operator-related factors, such as LCU tip position or angulation, could minimize the delivered R_E_ and subsequently compromise the degree of conversion [11,15]. However, whether the variation of radiant exposure delivered to the top surface (occlusal view) of a bulk-fill composite will alter the properties pertinent for the longevity of restorations of the resin is not yet clear. Many studies in the broader literature have been published concerning biofilm growth over conventional and bulk-fill composite [16,17,18,19,20,21], but only considering ideal curing settings. No articles have been found dealing with the cariogenic biofilm growth over bulk-fill composites under curing settings that simulated the most frequently performed light-curing procedures.

We present the effects of radiant exposure (RE) and radiant emittance performed in optimum and underperformed conditions on the degree of conversion (DC%), *Streptococcus mutans* biofilm formation, and surface roughness. The null hypothesis to be tested is that varying R_E_ delivered to bulk-fill composite would not affect DC%, *S. mutans* biofilm growth, and surface roughness.

## 2. Materials and Methods

### 2.1. Experimental Design

**Figure 1:**
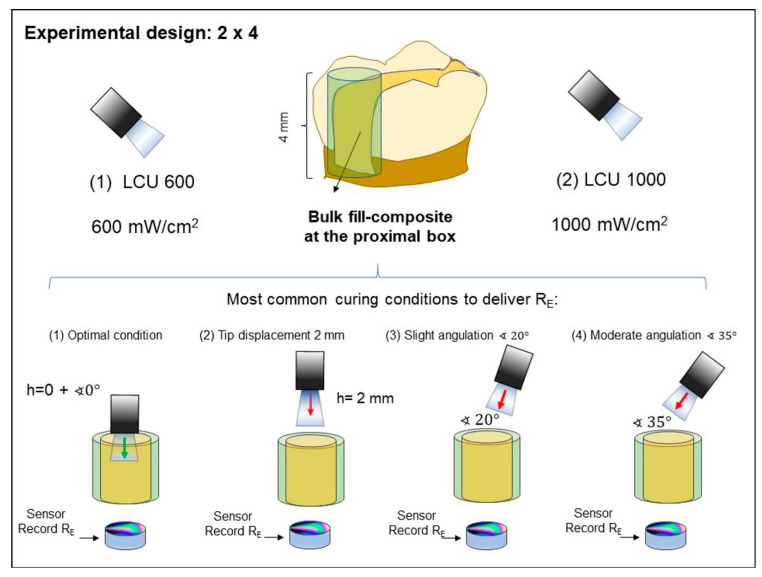
Schematic representation showing the most clinical conditions during the light-curing procedure, which was simulated in this experiment. The 4-mm increment was placed similar to that one placed in the proximal box of a Class II preparation. Then, the following simulated conditions: (**1**) optimal condition (no angulation or tip displacement), (**2**) tip-displacement (2 mm), (**3**) light tip angulation (α = 20°) and (**4**) light tip angulation (α = 35°) were performed, and the R_E_ values were recorded.

Two LCUs were used, LCU_600_ (Radii-cal, SDI Limited Victoria, Australia; standard curing mode, radiant emittance output provided of almost 689 mW/cm^2^) and LCU_1000_ (Valo grand, Ultradent Products Inc., South Jordan, UT, USA; standard curing mode; radiant emittance output provided of 1029 mW/cm^2^). These LCUs were representative of the most common radiance emittance found in commercially available LCU: 600 and 1000 mW/cm^2^, respectively. The output of each LCU was confirmed using a laboratory-grade NIST-referenced USB4000 Spectrometer (MARC: Managing Accurate Resin Curing; System, Bluelight Analytics, Halifax, NS, Canada). The curing technique was performed following four conditions: (1) optimal condition (no angulation or tip displacement), (2) tip-displacement (2 mm), (3) light tip angulation (α = 20°) and (4) light tip angulation (α = 35°) (Figure 1). These scenarios represent the optimal and underperformed curing techniques, which have been described as the most commonly performed conditions in dental practice [26]. After performing the light-curing procedure following these conditions, four different values were recorded, which are (1) radiant exposure (R_E_ in J/cm^2^), (2) the degree of conversion (DC%) on the top and bottom of specimens, (3) colony-forming units (CFU) of *S. mutans*, and (4) surface roughness.

A bulk-fill composite applied in a 4-mm increment was used in all tested conditions (3M ESPE Filtek Bulk Fill Posterior Restorative material, shade A2, St. Paul, MN, USA). According to the manufacturer, the bulk-fill composite contains silica, zirconia, zirconia/silica cluster, and ytterbium trifluoride filler particles (76.5 wt.% or 58.4 vol.%). It also contains aromatic dimethacrylate, urethane dimethacrylate, and 1,12-dodecanediol dimethacrylate in the comonomer blend.

### 2.2. Sample Preparation and Measurement of Radiant Exposure (R_E_) at the Bottom Surface of the Specimen

3D printed molds were designed to achieve good standardization during bulk-fill composites curing in the four different conditions, either with LCU_1000_ or LCU_600_ (Figure 1). The 3D printed molds (polylactic acid filament, melted Extrusion Modeling, 3D H800 Afinia printer, Chanhassen, MN, USA) were designed with an inner diameter of 7 mm and thickness of 4 mm. Before performing each condition, the R_E_ values were obtained with the LCUs placed directly on the sensor of a laboratory-grade NIST-referenced USB4000 Spectrometer (MARC: Managing Accurate Resin Curing; System, Bluelight Analytics, Halifax, NS, Canada). An assessment was made from the top of the empty mold to observe the RE obtained without the bulk-fill. Afterward, the R_E_ that was able to transmit through the bulk-fill thickness (n = 6) to the underlying sensor at the bottom of the composite cylinders was measured for each condition (Figure 1). The photoactivation was performed for 20 s using one of the two LED-curing units, LCU_600_ and LCU_1000_, at a radiant emittance output of approximately 600 or 1000 mW/cm^2^. All the composite cylinders were dried and stored at 37 °C for 24 h.

The total energy delivered to the specimen stated as radiant exposure (R_E_) was calculated according to the following Equation (1) [27]:

Equation (1):

(1) $$\frac{J\;\left(\mathrm{joules}\right)}{{\mathrm{cm}}^{2}}=\frac{\mathrm{mW}}{{\mathrm{cm}}^{2}}\times \;t\left(\sec\right)$$

where mW/cm^2^ is the radiant emittance or intensity from the LCU.

### 2.3. Degree of Conversion Analysis

Fourier transform infrared (FTIR) spectroscopy was used to assess the conversion level attained by the curing procedures. Bulk-fill samples were protected from additional lighting for 24 h at 37 °C after photoactivation [28]. The cylinders (n = 3) were embedded into epoxy resin and sectioned using a diamond saw (Accutom-5, Struers, Cleveland, OH, USA) to obtain three 0.4 mm thick slices parallel to the long axis of each cylinder. The slices were positioned over the platform of an IR microscope (Nicolet Continuum) coupled with an IR spectrometer (Nicolet 6700, ThermoFisher, Madison, WI, USA). Spectral data were obtained in the near-IR spectral region (NIR—from 4000 to 14,000 cm^−1^). The spectra data were obtained for each prepared slice corresponding from the top to bottom length of the bulk-fill cylinder. Spectra of the uncured composite were used to calculate the vinyl double bond conversion at each depth using the vinyl overtone peak area at 6165 cm^−1^ [29]. 2D maps of the degree of conversion as a function of depth were produced.

### 2.4. Quantification of S. mutans Biofilm on Bulk-Fill Composites

*S. mutans* (ATCC 700610, UA159; American Type Culture, Manassas, VA, USA) biofilms were initiated over the cured specimens’ bottom side as previously described [27,28,29,30] with some modifications. *S. mutans* was used as inoculum according to a protocol approved by the local institution.

In summary, 150 µL of *S. mutans* inoculum in brain heart infusion (BHI, Sigma-Aldrich, St. Louis, MO, USA)–glycerol solution (stored at −80 °C) was spread on Columbia blood agar (BBL, Becton Dickinson, Allschwil, Switzerland) and incubated for over 48 h. *S. mutans* colonies were resuspended in 5 mL of BHI broth and incubated overnight at 37 °C under the aerobic condition to the mid-log phase (OD_600_ = 0.9). The cured bulk-fill composite samples (n = 6) were sterilized via ethylene oxide gas and placed in a well of a 24-well plate containing sterile BHI containing 5% sucrose [27]. Next, 120 μL of overnight cultures of *S. mutans* (10^8^ CFU/mL) were inoculated in each well. The inoculation of each BHI-containing recipient was performed only once on the first day, and the bulk-fill composite samples were transferred to a fresh medium every day for 14 days. Each BHI-containing well was streaked onto a new fresh BHI agar media plated and incubated at 37 °C in an atmosphere of 10% CO_2_ for 24 h to evaluate the purity.

For biofilm viability assessment, the biofilms formed on the bulk-fill composite samples were collected, serially diluted with 0.9% sodium chloride (NaCl) solution and plated in triplicate on BHI agar. After 48 h at 37 °C in a 10% CO_2_ atmosphere, representative colonies with typical morphology of *S. mutans* were counted and expressed as CFU/composite.

### 2.5. Morphologically Evaluation of S. mutans Biofilm over Bulk-Fill Composites

After the biofilm formation on bulk-fill composites for 14 days, one sample from the control group and another from the moderate angulation condition were prepared for a qualitative analysis via scanning electron microscopy (SEM, Quanta 200, FEI, Hillsboro, OR, USA). First, a fixation and dehydration process was performed with Karnovsky’s fixative and alcoholic solution, sputtering with gold/palladium. Then, the samples were examined at 200× and 10,000× magnification at an accelerating voltage of 20 kV.

### 2.6. Surface Roughness of Bulk-Fill Composites after Exposure to S. mutans Biofilm

The surface roughness (Ra, μm) of bulk-fill composites (n = 6) after *S. mutans* biofilm formation for 14 days was measured. Each sample’s bottom was analyzed for this assay before and after the *S. mutans* biofilm formation using a surface roughness measurement instrument (Surftest SJ-310, Mitutoyo America, Aurora, IL, USA). Five measurements of each bulk-fill sample were performed using the stylus tip (5 µm) at a constant speed of 0.5 mm/s, a force of 4 mN, with a 0.25-mm cutoff value, and 1.5-mm tracing length [27]. Ra (ΔRa) variation was calculated by measuring the difference between the final and initial Ra.

### 2.7. Morphologically Evaluation of Bulk-Fill Composites Surface after Exposure to S. mutans Biofilm

After the exposure to *S. mutans* biofilm, one sample from the control group and another from the moderate angulation condition were prepared for a qualitative analysis of their surface via SEM (Quanta 200, FEI, Hillsboro, OR, USA). The samples were coated via sputtering with gold/palladium and analyzed with a magnification of 200× and 10,000× at an accelerating voltage of 20 kV.

### 2.8. Statistical Analysis

Statistical evaluations were performed with Sigma Plot (Sigma Plot 12.0; SYSTAT). The Shapiro–Wilk test was applied to verify if the data were normally distributed. Results were compared using two-way analysis of variance (ANOVA) and Tukey’s test (α = 0.05). A linear Pearson correlation assessed the correlations between R_E_ and the outcomes of each test.

## 3. Results

**Figure 2:**
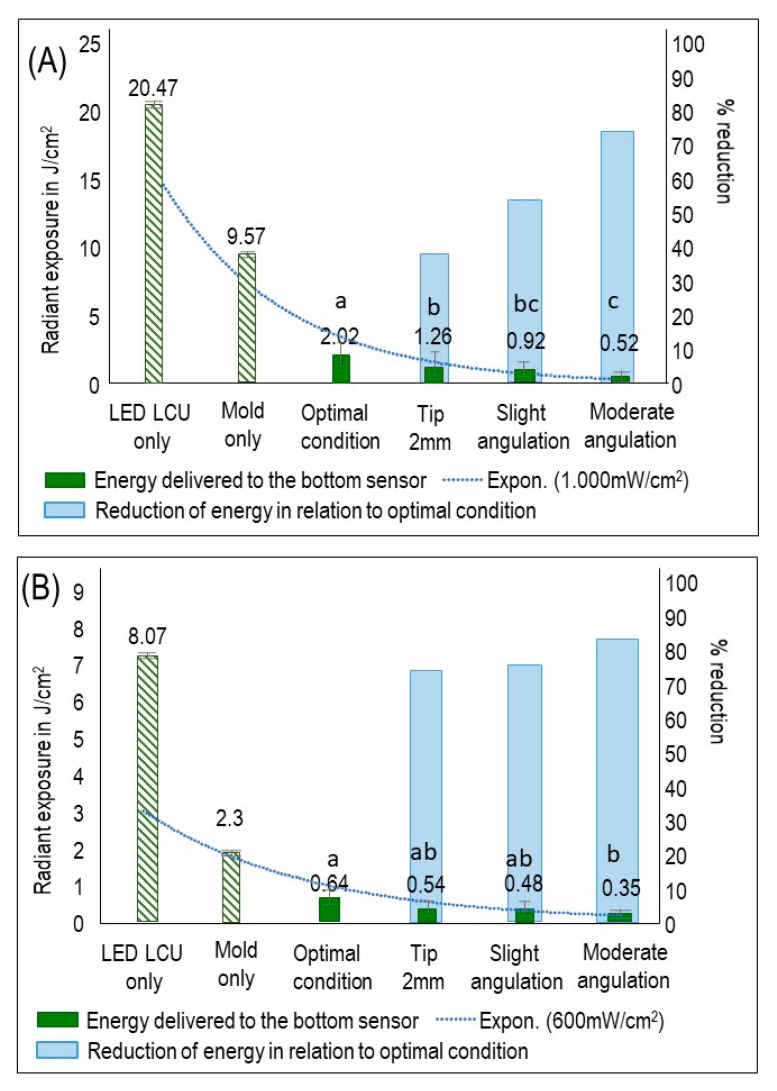
R_E_ values that reached the sensor and the percentage of R_E_ reduction in the underperformed conditions compared to optimal condition (mean ± sd; n = 6). (**A**) R_E_ values and the percentage of R_E_ reduction using LCU_1000_ with an output of 1000 mW/cm^2^. (**B**) R_E_ values and the percentage of R_E_ reduction using LCU_600_ with an output of 600 mW/cm^2^. On the first *y*-axis, the barplot demonstrates radiant exposure (R_E_) expressed in J/cm^2^. On the second *y*-axis, the barplot demonstrates the percentage of R_E_ reduction. The dotted line illustrates the reduced R_E_ for all groups concerning R_E_ delivered to the sensor by applying the LCU directly on the sensor. Values with different letters are significantly different from each other (*p* < 0.05).

Figure 2 shows the R_E_ values for LCU_1000_ (Figure 2A) and LCU_600_ (Figure 2B). In general, LCU_1000_ revealed a higher and significant R_E_ value (2.02 mW/cm^2^) than LCU_600_ in both optimal and underperformed conditions (*p* < 0.05; power of analysis 100%). In LCU_1000_, the R_E_ value of the optimal condition was significantly higher than the other underperformed conditions (*p* < 0.05) (Figure 2A). For LCU_600_, the R_E_ of optimal condition (0.64 mW/cm^2^) was not significant (*p* > 0.05; power of analysis 100%) compared to 2 mm tip displacement (0.54 mW/cm^2^) and slight angulation (0.48 mW/cm^2^), but then significant compared to moderate angulation (*p* < 0.05) (Figure 2B). In Figure 2, the first *y*-axis represents the R_E_ values, while the second *y*-axis represents the reduction of R_E_ in the three underperformed conditions compared to the optimal conditions. The dotted line illustrates the decay in R_E_ that reached the sensor in all groups. In LCU_1000_, the reduction in R_E_ in the underperformed conditions ranges from 37.6 to 74.2% compared to the optimal condition. While in LCU_600_, the reduction was observed between 15.6 and 45.3%. The influence of the less than optimal light conditions (F = 10.48, *p* < 0.001) and irradiance output of LCU (F = 10.30, *p* = 0.0024) on the radiant exposure (R_E_) were considerable.

**Figure 3:**
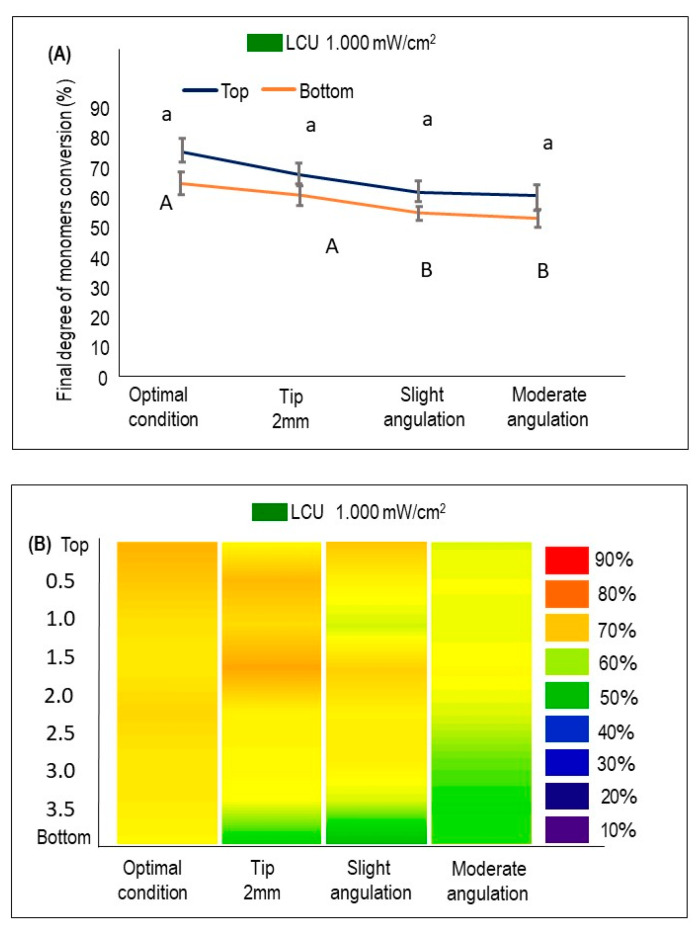
The %DC using LCU_1000_ with an output of 1000 mW/cm^2^ at the top and bottom of BF composites cured optimally or following the underperformed conditions (mean ± sd; n = 3). (**A**) The %DC values at the top and bottom of each condition. Lower case letters compare the %DC at the top of the specimen, while capital case letters compare the %DC at the bottom. Dissimilar letters are significantly different from each other (*p* < 0.05). (**B**) The %DC is measured at different depths from top to bottom and visualized by the heat maps. The color bar (right) illustrates the visual representation of the %DC corresponding to the colors seen.

Figure 3 demonstrates the DC% at the bottom and the top of the sample when the LCU_1000_ was used (mean ± sd). In LCU_1000_, no significant difference was found among the DC% results on the samples’ top when the curing conditions were compared (Figure 3A). However, a significant decrease in DC% at the bottom of the samples was observed when slight and moderate angulations were performed (*p* < 0.05). The DC% was reduced by around 10% in moderate angulation conditions compared to the optimal condition when the bottom surfaces were examined. In Figure 3B, the heat maps of the average DC% of samples cured with LCU_1000_ were investigated at different depths from the top surface of each sample to the bottom. Reducing DC% towards the bottom of the specimen is visualized by increasing the cold colors. A trend in dropping the DC% was observed for groups subjected to angulations with a predominance of a green color corresponding to 50–60% of conversion.

**Figure 4:**
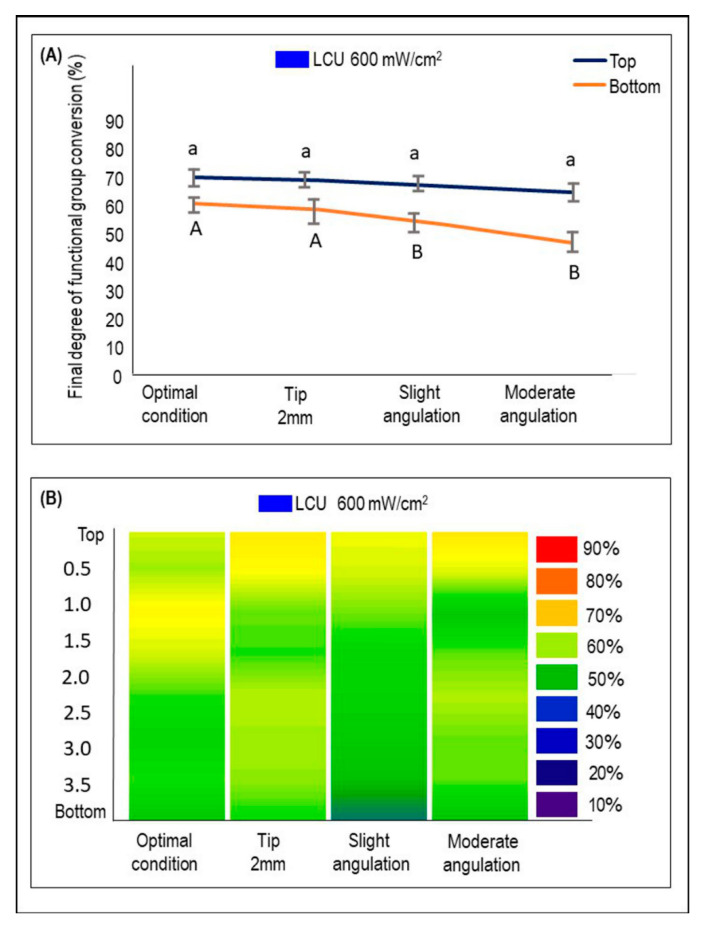
The %DC using LCU_600_ with an output of 600 mW/cm^2^ at the top and bottom of BF composites cured optimally or following the underperformed conditions (mean ± sd; n = 3). (**A**) The %DC values at the top and bottom of each condition. Lower case letters compare the %DC at the top of the specimen, while capital case letters compare the %DC at the bottom. Dissimilar letters are significantly different from each other (*p* < 0.05). (**B**) The %DC is measured at different depths from top to bottom and visualized by the heat maps. The color bar (right) illustrates the visual representation of the %DC corresponding to the colors seen.

Figure 4 demonstrates the DC% at the bottom and the top of the sample when the LCU_600_ was used (mean ± sd). No significant difference was found among the DC% results on the samples’ top when the curing conditions were compared. The top and the bottom difference was 8 to 11% for the groups subjected to slight and moderate angulations, respectively. When the bottom surfaces of the samples cured with different conditions were examined, the DC% was significantly reduced in the slight and moderate angulation conditions compared to the other groups (*p* < 0.05). The DC% reduction is represented in Figure 4B. The transition of the colors to dark and light green indicates a decrease in the DC% values. Comparing the heat maps of LCU_1000_ and LCU_600_ shows that LCU_1000_ is associated with a higher DC% than LCU_600_. Pearson’s correlation coefficient indicated strong correlation between R_E_ and DC% values (r = 0.611; *p* = 0.0047).

**Figure 5:**
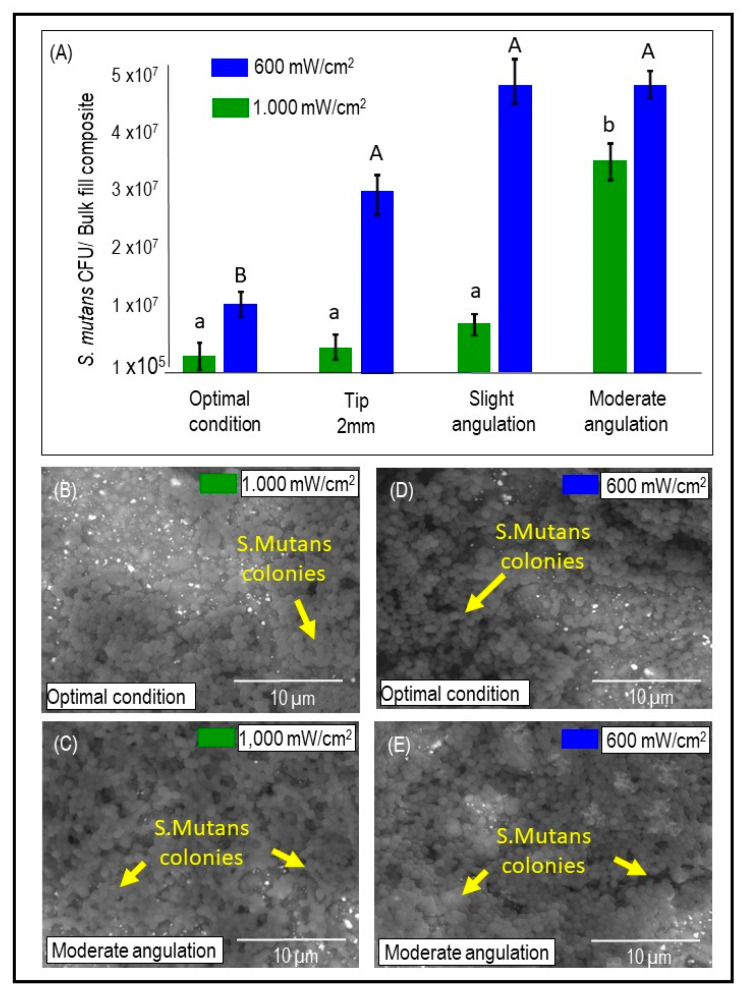
The quantitative and qualitative analysis of the *S. mutans* biofilm grown over the bulk-fill (BF) composites cured in different conditions. (**A**) *S. mutans* colony forming units (CFU) growth over the BF composites for each LCU output and each light-curing condition (mean ± sd; n = 6). (**B**–**E**) Representative SEM images showing bacterial adhesion and biofilm formation on the bottom surfaces of composite cylinders for LCU_1000_ (**B**,**C**) and LCU_600_ (**D**,**E**) under optimal and moderate angulation conditions. Note arrows point to the *S. mutans* colonies. Capital letters compare LCU_600_, while lower case letters compare LCU_1000_. Dissimilar letters are significantly different from each other (*p* < 0.05).

Figure 5A illustrates *S. mutans* colony-forming units counting expressed by CFU/ composite for both the LCUs output and the four tested conditions (mean ± sd). In LCU_1000_, R_E_ had no significant effect regarding the CFU of *S. mutans* except when the LCU tip is moderately angulated (*p* < 0.05; power of analysis 100%). While in LCU_600_, the optimal condition R_E_ was associated significantly with less CFU than the other three underperformed conditions (*p* < 0.05; power of analysis 100%). The Pearson correlation between R_E_ and *S. mutans* biofilm formation (*p* = 0.0027; r = −0.49) demonstrated an inverse relationship between the two factors: lowered radiant exposure values were associated with high *S. mutans* biofilm formation. In addition, moderate angulation of the LCU tip was associated with a significant increase in *S. mutans* viability (*p* < 0.05). In Figure 5B–E, representative SEM images demonstrate higher bacterial adhesion and biofilm formation in moderately angulated bulk-fill composite samples than optimal conditions for both LCU_1000_ and LCU_600_.

**Figure 6:**
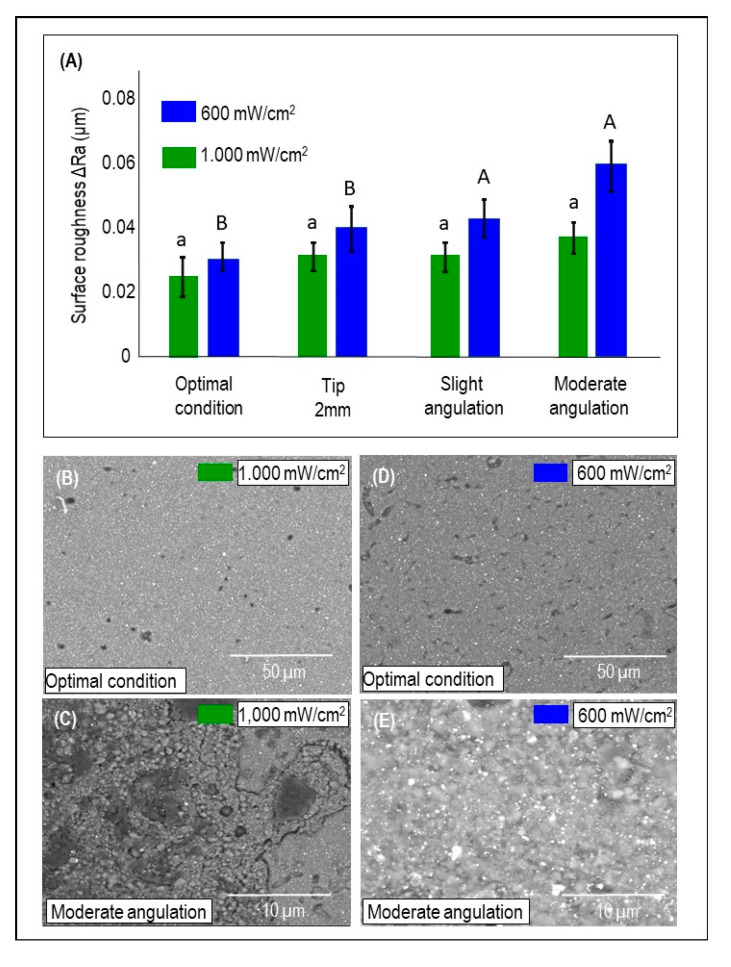
The topography changes of the bulk-fill (BF) composites cured in different conditions. (**A**) The mean and standard deviation of ΔRa values considering the underperformed light curing conditions and the two-radiance emittance output (mean ± sd; n = 6). Capital letters compare LCU_600_, while lower case letters compare LCU_1000_. Dissimilar letters are significantly different from each other (*p* < 0.05). (**B**–**E**) Representative SEM images of the BF composite surface under control and moderate angulation following the output of 1000 mW/cm^2^ (**B**,**C**) and LCU600 (**D**,**E**).

Figure 6A demonstrates the mean and standard deviation of ΔRa values for the light curing conditions using either LCU_1000_ or LCU_600_ (mean ± sd). The radiance emittance output (*p* = 0.0041) and less than optimal light conditions (*p* = 0.0207) have a statistical effect using two-way ANOVA, although no interaction was observed (*p* = 0.271). LCU_1000_ did not show any significant difference concerning ΔRa for all curing conditions, but LCU_600_ demonstrated a significant difference between the optimal condition and slight and moderate angulation conditions (*p* < 0.05; power of analysis 100%). In Figure 6B–E, SEM images of the bulk-fill composite surface are illustrated when optimal and moderate angulations were performed using either LCU_1000_ or LCU_600_. The most noticeable difference was found when using the radiance emittance output of LCU_600_ with a moderate angulation (0.062 um). In addition, superficial degradation and exposed fillers resulting from resin matrix loss were observed with moderated angulation (Figure 6C,E).

## 4. Discussion

In this study, improper polymerization and low DC% of bulk-fill composites are associated with more biofilm growth and increased roughness. Furthermore, these complications were intensified with incorrect light-curing techniques such as tip displacement and improper angulation. Frequently, in vitro studies are performed in optimal conditions where most of the variables are well-controlled. Nevertheless, the clinical setting situation is different, as many clinical variables may complicate the curing procedure.

Variables such as the tooth’s position inside the mouth, the anatomy of the tooth, the position of the placed restoration, and moisture challenges may compromise the R_E_ delivery [11]. Class II cavity preparation represents a clinical situation where the delivery of adequate R_E_ is challenging as cavity walls. Cusp tips may interfere with closer tip placement—consequently, light transmission, especially to the bottom portion of the gingival floor [11]. Additionally, operator-related factors significantly impacted the delivered R_E_ as previously reported [11,14,15] and demonstrated in this study.

Two radiant emittance outputs of approximately 600 or 1000 mW/cm^2^ [31] delivered by two distinct LCUs were used to conduct the present study. The spectral output of these lights and the beam dispersion with distance and light tip diameters are different. Radii-Cal is a mono-wave LED LCU, while VALO grand is a multi-wave LED LCU with two predominant peaks, in which one has a shoulder extending the emittance wavelength to almost three peaks [32]. The rationale behind their selection for this research was based on using two commonly used LCU under clinical situations by dentists.

For maximum curing, 50% to 60% functional group conversion is expected [11,12]. In a systematic review of 21 studies [31], eleven studies demonstrated acceptable DC% values higher than 50% for bulk-fill composites; eight studies demonstrated material-dependent results, and two studies reported unacceptable bottom/top hardness ratio [33,34]. Most studies reported acceptable DC% when using an LCU that generates ≥1000 mW/cm^2^, which also was found in our study as the amount of R_E_ delivered to the sensor was higher using LCU_1000_. It was observed here that under an optimal curing condition, the DC% achieved 70% using the LCU_1000_. However, when the LCU_600_ was used, DC% ranged from 50 (bottom) to 70 (top). These results suggest that using an LCU with ≥1000 mW/cm^2^ radiant emittance is preferable when curing bulk-fill composites. This fact is reinforced by the unacceptable bottom/top hardness ratio and low polymerization using LCU with radiant emittance values of 700 and 800 mW/cm^2^ [33,34].

The radiant exposure of 16 J/cm^2^ is often considered the threshold value of radiant energy influx required for maximum curing of a 2-mm increment [35]. This can be delivered by a 20 s exposure to an LCU emitting 600 mW/cm^2^. The degree of conversion also varies according to material-related factors such as the translucency, and filler content may affect the amount of required energy to achieve acceptable polymerization [11]. Here, the recorded R_E_ reaching the underlying sensor represents the R_E_ energy that reached the bottom layer of bulk-fill composites.

Previous studies indicate that sufficient polymerization could be achieved when a radiant exposure of 0.7–1.5 J/cm^2^ is delivered to the bottom layer [36,37,38]. When our findings are compared with this range, the results indicate the proper delivery of radiant exposure for polymerization. Note that the optimal condition using LCU_1000_ achieved a R_E_ of 2.02 J/cm^2^. The improper simulated curing conditions achieved values between 0.52 and 1.26 J/cm^2^, compromising the polymerization quality. The use of LCU_600_ (with 600 mW/cm^2^) as output greatly reduces the R_E_ values (range values 0.35–0.64 J/cm^2^) under incorrect curing conditions. Additionally, the low percentage of conversion found for this group may reflect the detrimental effect of using LCU_600_ with 600 mW/cm^2^.

*S. mutans* is recognized as one of the major species related to dental caries. This virulent oral pathogen has acidogenic and aciduric properties and an enhanced ability to attach to surfaces and survive over different substrates [39,40]. Our work demonstrated higher *S. mutans* biofilm formation over bulk-fill composites cured with underperformed conditions. Moderate angulation demonstrated the more prominent amount of *S. mutans* biofilm and surface roughness. The discovered detrimental outcome could be related to leached uncured monomers that facilitate bacterial adhesion and penetration through the bulk-fill composites [41]. Several studies emphasized *S. mutans* biofilm’s role in composite degradation and compromising the integrity and smoothness of the surface [39,40,41,42].

Esterases are essential virulence factors in the pathogenicity and cariogenicity of bacterial species [39]. *S. mutans* esterase virulence gene can catalyze the uncured monomers, causing further degradation, leading to bacterial colonization at the margin and recurrent caries [43]. LCU_600_ generally demonstrated more biofilm formation than LCU_1000_, which could decrease R_E_ value and DC%. The biofilm was maintained for 14 days to allow a mature cariogenic biofilm that resembles the dental caries process in the oral cavity.

Additionally, uncured monomers sub-products are mainly observed within seven days with similar characteristics compared to sub-products released after 30 days [44,45]. The increased Ra values found, especially for moderate angulation conditions, may clinically jeopardize the treatment outcome. Different bulk-fill composites formulations, such as the quantity of inorganic filler and the blend composition, could be interesting to evaluate biofilm accumulation under these photocuring conditions. Moreover, we used a single-specie biofilm model. Although the biofilm was grown for an extended period, a high-challenge multispecies biofilm model may accelerate the bulk-fill composites’ degradation process and surface changes, mainly those receiving lower R_E_.

This study’s overall outcomes found resonance in our group’s earlier work when Maktabi et al. [27] showed a striking prejudicial effect of radiant emittance of 600 mW/cm^2^ and incorrect curing techniques biofilm growth over conventional RBCs. In this cited study, similar methodology and assessments were applied, which allows us to draw a comparative profile. The negative influence of low R_E_ on increased *S. mutans* growth and reduced DC% was shown, as expected, similar to our results. Under the same simulated curing conditions (tip displacement and incorrect angulations), the radiant exposure delivered to a 2-mm increment in conventional RBCs led to R_E_ reduction values varying from 49.4 to 73.5% in relation to the control group. The difference in DC% between the top and the bottom of 2-mm conventional RBCs discs varied from 13 to 21% for 1000 mW/cm^2^ and 29 to 53% for LCU_600_.

Here, the curing conditions applied to 4-mm bulk-fill increment showed varied R_E_ from 15.6% to 82.9% compared to the control group. Our results showed a difference in DC% between the top and bottom of the bulk-fill composite ranging from 10 to 20% for 1000 mW/cm^2^ and 8 to 11% for 600 mW/cm^2^. This finding is essential to guide an interpretation of different materials under similar conditions. Most importantly, it can suggest that under 600 mW/cm^2^, the bulk-fill composite has shown more minor variation to the detrimental effects of incorrect curing procedures. In both studies, an increased *S. mutans* biofilm formation was significant for angulated and distant curing procedures observed via colony-forming unit counting and SEM analysis.

The limitations of this study include the use of only one commercial type of bulk-fill composite. Recently, an investigation was performed with different bulk-fill composites to analyze their roughness, surface free energy, and adhesion of *S. mutans* or *Streptococcus mitis.* [46] In this study, the roughness was not different among the four commercially available composites (Sonic Fill-2 (Orange County, CA, USA), Filtek BulkFill (Saint Paul, MN, USA), Admira Fusion X-tra (Cuxhaven, Germany), and Beautifil Bulk Restorative (Shofu, San Marcos, CA, USA)). However, there were significant differences in their contact angle and surface free energy, which the authors reported as a feature that could lead to different microorganisms’ adhesion in an actual clinical situation [46].

Therefore, further studies could reproduce similar experiments using varied LCU tip displacement or angulation and different commercial bulk-fill composites. The variations in material components, amount of filler, and photoinitiators may provide different results from one product to another [8]. Another limitation is the use of a single species biofilm [23]. It is more clinically relevant to use a multispecies biofilm to investigate the effect of biofilm accumulation in bulk-fill composite degradation. It is expected when using such a complex biofilm model that the amount of degradation and surface changes will be higher compared to what was found in this study [24].

The findings here reported are novel, primarily in the context of exploring the bacterial response to varied R_E_ intended to cure bulk-fill composites. Moreover, we spent efforts to understand the potential risks of triggering a cascade of possible events that could compromise restoration’s long-term performance in the oral environment. Thus, dentists should always be attentive to optimizing the curing procedures, especially when using bulk-fill composites in critical situations as deep proximal cavities.

## 5. Conclusions

Based on our in vitro outcomes, inadequate polymerization of bulk-fill composites could be associated with more biofilm accumulation and surface topography changes. Insufficient polymerization was triggered by poor curing conditions such as LCU tip displacement and angulation, along with the use of LCUs that induce 600 mW/cm^2^ output.

## Data Availability

The data set generated and analyzed in this study is available upon reasonable request to the corresponding author.
